# Effect of emotional valence on episodic memory stages as indexed by event-related potentials

**DOI:** 10.4236/wjns.2013.34034

**Published:** 2013

**Authors:** Marc E. Lavoie, Kieron P. O’Connor

**Affiliations:** 1 Centre de Recherche, Institut Universitaire en Santé Mentale de Montréal, Montréal, Canada; 2 Département de Psychiatrie, Université de Montréal, Montréal, Canada

**Keywords:** Emotion, Memory, Familiarity, Recollection, Old/New Effect, Event-Related Potentials

## Abstract

Several investigations have shown that emotional events show superior recall than non-emotional ones. However, the cortical mechanisms underlying the episodic recall of emotional scenes are still poorly understood. Our main aim was to compare the magnitude of the Event-Related brain Potentials (ERP) old-new effect related to emotionally unpleasant, pleasant and neutral photographic images. As expected, correct recognition of all types of images elicited three topographically distinct ERP components sensitive to the classical old-new recognition effect. The results revealed that the behavioral performances were mainly sensitive to arousal, while the ERP old/new effect over posterior regions (300 – 1000 ms) was exclusively affected by unpleasantness. A later component (1000 – 1400 ms) showed an inverted old/ new effect at parietal sites, which was also sensitive to unpleasantness. These results imply that ERP reflecting episodic conscious recollection and post-retrieval monitoring are clearly affected both by valence and arousal.

## 1. INTRODUCTION

The presence of an emotional context plays an important role in memory, affecting both encoding and retrieval processes [1,2]. One way to study such factors is to relate recognition memory to the presence of emotionally evocative stimuli. Emotional stimuli can be represented by a two-dimensional space with one axis defined by the valence (from unpleasant to pleasant) and the other axis by arousal (from calming to exciting) [3]. Rather than assuming independent discrete emotional states (*i.e.* happiness, anger, etc.), a dimensional view of emotion assumes these two primary dimensions encompass the spectrum of emotional behavior. These dimensions of emotional arousal and valence have been operationalized through studies of the recall of photographic images depicting complex scenes (landscapes, animals, crime scenes, erotica, mutilations, car accident, etc.). Bradley, Greenwald, Petry and Lang [4] found that photographic images rated as highly arousing were recalled more accurately than low-arousal images. High-arousal images, encoded earlier in the experiment, produced faster reaction times than their low-arousal counterparts, with no additional effects due to valence. However, unpleasant pictures (high and low-arousal) delayed reaction times for the new pictures not encoded earlier. This finding suggests that both dimensions of arousal and valence are salient during initial encoding.

Indeed, brain-imaging studies have supported the hypothesis that short-term memory is enhanced in the presence of unpleasant compared to neutral or pleasant pictorial scenes [5]. An adaptation mechanism may favor aversive images through enhancement of retrieval and consolidation in memory, perhaps via the mediating role of the amygdala or other networks sustaining recognition memory [2]. With Event-Related Potentials (ERP), it has been shown that ongoing emotional stimuli are evaluated at several points in the information processing stream and the affective content could interact with many processes within a few hundred milliseconds after stimulus onset [6–9].

In recognition memory paradigms which included non-emotional material, it has been consistently shown that items previously presented (old) elicit a larger parietal ERP amplitude than the items not (new) previously presented [10–12] generating three topographically dis tinct “old/new” effects. First, an early (300 – 500 ms) bilateral, frontal effect occurs when the access to perceptual and conceptual information related to test items is facilitated [10,11,13,14]. Secondly, a left parietal old-new effect (500 – 1000 ms) shows larger amplitude for deeply than to superficially encoded items. This parietal deflection reflects conscious recollection [15–19]. Following the parietal deflection, a late old-new effect (1000 – 1400 ms) normally arises just after the correct identification of the stimulus. Recent investigations propose that this effect is sensitive to the extrinsic (*i.e.* background) context in face recognition [20] and to a post-retrieval monitoring mechanism [19,21].

In the field of emotional memory, Maratos, Allan & Rugg [22] investigated these three recognition stages in relation to emotionally negative and neutral words. They showed that the left parietal old-new effect for negative words was smaller than that elicited by neutral words. Maratos *et al*. suggested that emotionally valenced words influence recognition memory mainly because of their higher levels of semantic cohesion, which leads to a tendency for false recollection of new negative items, diminishing the parietal old-new effect. In a similar study, Windmann and Kutas [23] proposed that emotional words could induce a propensity to respond “old” to a negative rather than to a neutral item, whether on not the item is actually old or new. In their investigations, the old-new ERP difference for correctly recognized items, was largely unaffected by negative words. So, regardless of emotional valence, the ERP associated with old words was characterized by a widespread positivity relative to that for correctly rejected new items. However, ERPs to hits (old) and false alarms (new) revealed a valence effect. While neutral items showed a large old/new difference, negative items did not. Windmann *et al*. [23] proposed that the frontal cortex might be responsible for relaxing the retrieval criterion for negative words so as to ensure that emotional events are not as easily forgotten as neutral events.

Two complementary conclusions emerge from these two ERP investigations. First, episodic retrieval is affected by the negative emotional valence of a stimulus. Second, the emotional negative valence seems to affect specific stages of memory processing while leaving other processes relatively intact. However, as noted earlier, ERP studies are in the unique position to inform brain imaging studies contrasting pleasant and unpleasant valence with neutral images by monitoring relevant recognition memory stages.

The goals of the present study were twofold. The first aim was to investigate whether recognition performances are affected by the presentation of emotional images when controlled for valence and arousal level ratings. The second goal was to consider whether the three recognition memory stages, as indexed by ERPs, are affected equally by emotional valence and arousal. The comparison between equally arousing pleasant and unpleasant images would clarify the separate influence of emotional valence and emotional arousal on specific memory stages. Based on previous data obtained with similar emotional images [4,24], we hypothesized that both valence and arousal would have an impact on recognition performances. Electrophysiologically, we also predicted that unpleasant images would mainly affect conscious recollection and, thus, reduce the left parietal old-new effect [22].

## 2. METHOD

### 2.1. Participants

Twenty right-handed female participants with normal or corrected-to-normal vision were selected. The mean age of the group was 25 years (range = 19 – 48 years old) with an average schooling of 16 years. Participants were administered the Raven test of non verbal intelligence and the California Verbal Learning Test (French version-CVLT) in order to validate and ensure normal functioning in general intelligence and verbal memory. All participants scored within the normal range according to published norms. All participants were recruited by announcements in the local media and were screened initially by telephone for suitability in terms of geographical accessibility, motivation to attend and absence of psychiatric or medical history. Only female participants were included in order to homogenize variance of our sample, since earlier studies demonstrated gender differences in affective processing and reported greater reactivity in women to aversive than pleasant pictures [24, 25].

### 2.2. Experimental Setting

On arrival at the laboratory, participants read and signed an informed consent form and received psychometric testing (CVLT and Raven test). EEG recordings were made in a dimly lit room where the participant was seated in an adjustable chair in front of the computer monitor. The recording room constituted a separate corner of a larger room in which the experimenters, amplifiers and computers were located. One experimenter gave instructions, while another experimenter was assigned to control the experiment with constant visual monitoring of the participant by means of a video camera. The nylon electrode cap (Electro Cap International), electro-oculogram and mastoid references were installed within 30 minutes. A one-minute resting baseline was recorded at the beginning of the experiment to facilitate laboratory adaptation.

#### 2.2.1. Stimuli Selection

The emotional materials were photographic images from the International Affective Picture Systems (IAPS: Center for the study of emotion and attention [CSEA-NIMH], 1998), a standardized collection of images gathered from a wide variety of emotional and semantic categories.

A total of 150 photographic images were chosen and classified into three groups, based on the arousal and valence estimation from the IAPS normalization (50 unpleasants, 50 neutrals and 50 pleasants). The stimuli for the study phase included a total of 75 images. For the test phase, the lists included the 75 images of the study phase (old), plus 75 images that had not been presented (new) before. The images selected were classified into three basic categories, based on the IAPS female ratings of valence [unpleasant = 1 – 3; neutral = 4 – 6; pleasant = 7 – 9]. These 150 images (25 trials by 2 old/new response types by 3 valence categories—pleasant/unpleasant/neutral) were presented in different image orders to counterbalance effects due to sequence. In addition, for half of participants, the old/new order of presentation was inverted. Mean normative valence ratings from the IAPS were significantly different between valence estimation (F(5,144) = 353.02; p < 0.001). Multiple comparison post hoc tests (BONFERRONI) revealed that the *valence* was significantly different between neutral and unpleasant (p < 0.001), between neutral and pleasant (p < 0.001) and between unpleasant and pleasant (p < 0.001). These images also contained significant differences on *arousal* ratings (F(5,144) = 18.11, p < 0.001) and *post hoc* tests revealed that arousal ratings were significantly different between neutral and unpleasant (p < 0.001), between neutral and pleasant (p < 0.001), but not between pleasant and unpleasant images (p = 0.63). Finally, there were no significant differences between old and new categories across valence or arousal values (all p’s > 0.36). In each emotional category, the images contained the same basic attributes (scenes including humans, animals, in-animate objects or landscapes) across old and new category in order to preserve coherence across recall conditions.

The images were presented one at a time on a 17 SVGA monitor (Viewsonic), for a fixed duration of 4000 ms, at a distance of 90 cm calculated from the nose to the center of the computer screen with a 5 degree angle. They were presented at a resolution of 640 × 480 pixels in 256 colors without distortion between image presentations. The inter-trial interval (ITI) was fixed at 2000 ms during which a red and white checkerboard image appeared (IAPS #7182). This red and white checkerboard image informed the participant to fixate on a point between picture presentations and reduce the eye movements. This procedure also helped to reduce the after image effect, which occurred during presentation of a white blank background in our previous pilots.

#### 2.2.2. Experimental Procedure

The experimental session began with a study phase during which the participants were instructed first to fix their gaze on a red and white checkerboard screen while waiting for the next images to appear. At that point, participants were told that a series of image presentations would be presented and that they should attend to each picture the entire time it appeared on the screen without giving any response. A short retention interval of 10 minutes was allowed between the study and the test phase. In the test phase, images were projected for the same duration and ITI as for the study phase. The participants were instructed to detect the images that were presented (*old*) during the study phase by a button press and also to identify the images that were not present (*new*) during the study phase by pressing another button. The reaction times were obtained with a three button device placed in front of the subject. They were instructed to emphasize both speed and accuracy in their responses. The emotional evaluation based on the Self Assessment Manikin (SAM) was administered after the ERP experimentation and the participants rated, by a paper and pencil response, each of the 150 images presented in a booklet. Previous brain imaging studies using emotional photographic images have shown that task instructions, prompting preparation for the processing of the evocative images, are susceptible to affect neural activity [26]. So, for both study and test phases, participants were not informed about the emotional value of the images beforehand in order to minimize emotional expectancy before the task and the paper and pencil was done post-test to keep the emotional nature of the task implicit during the experimentation.

### 2.3. EEG Recordings and ERP Extraction

The EEG was recorded from 26 tin electrodes mounted in an elastic nylon cap (Electro-Cap International Inc.) only during recall (test phase). The scalp electrodes were placed according to the guidelines for standard electrode position by the American EEG Society [27] at F7, F8, F3, F4, Fz, FC3, FC4, T7, C1, C2, C3, C4, Cz, T8, TP7, CP3, CP4, TP8, P7, P3, Pz, P4, P8, O1, Oz and O2. All electrodes were referenced to linked mastoids and their impedances were kept below 5 KΩ. The Electro-oculograms (EOG) was recorded using four 9-mm tin external bi-polar electrodes for horizontal and vertical movements. For the horizontal EOG, electrodes were placed at the outer canthus of each eye and for the vertical EOG at infra and supra-orbital points at the left eye, aligned with the pupil looking straight. A bioelectric analog amplifier model ISS3-32BA (SAI-InstEP) amplified the EEG signals (EOG gain = ±10,000 and EEG gain = ±20,000) with a band-pass between .01 and 30 Hz. The EEG was recorded continuously at a sampling rate of 250 Hz and averaged offline in a time-window beginning at 100 ms before and until 1900 ms after picture onset. The EOG artifact contained in the EEG were corrected with a dynamic multiple regression in the frequency domain [28]. The regressions were applied using the horizontal and the vertical EOG activity subsequently. After EOG corrections, all remaining epochs with a voltage exceeding ±100 uV and clippings due to saturation or blocking of the amplifiers were eliminated automatically during the averaging procedure. On average, 2.5 trials per condition were rejected, after EOG corrections, because of the remaining artifacts (range = 0 – 5 trials). To exclude a possible residual effect of the EOG on the EEG and ERPs, ANOVAs, applied to the number of artifact rejected, failed to show any significant effect across response type and emotional valence conditions (all p’s over .30). A second ANOVA applied on the two EOGs separately also failed to reach any statistical significance according to response type or valence (all p’s over .10). A minimum amount of 16 trials free of both errors (false alarms and misses) and artifacts were included in the ERP averaging, which is comparable to the criteria used in similar ERP experiments [22,23,29–31]. Finally, four time windows were defined as the early (300 – 500 ms), middle (500 – 700 ms, 700 – 1000 ms) and late latencies (1000 – 1400 ms). Our experimental hypotheses were tested using the mean amplitudes of the ERP detected within the temporal windows as defined in previous recognition memory research [15,32].

### 2.4. Statistical Analyses

The behavioral data comprised the subjective affective ratings, median Reaction Times (RT), old/new discrimination accuracy (Pr), hits, False Alarms (FA) and response bias (Br). The Pr was computed by the following subtraction: Pr = Hit-FA where “Hit” represented the probability of “old response’’ to an old item and “FA” (False Alarm), the probability of “old response” to a new item. The Br was calculated on the basis of the FA and Pr as: Br = FA/(1 Pr) according to the two-high threshold theory (Snodgrass & Corwin, 1988). Mean ERP amplitudes for each time-window were collapsed across electrode sites to form the anteriority (anterior vs posterior) and the hemispheric (left/right) factors. The anterior electrodes were composed of F7, F3, FT7, FC3, T3, and C3 for the left hemisphere and F8, F4, FT8, FC4, T4, and C4 for the right hemisphere. The posterior electrodes included TP7, CP3, T5, P3 and O1 for the left hemisphere and TP8, CP4, T6, P4 and O2 for the right hemisphere. The behavioral and ERP data were submitted to a repeated measure ANOVA (SPSS-Windows ver 10.0). The behavioral data was submitted to an ANOVA with RESPONSE TYPE (with two levels; hits/correct rejection), and EMOTIONAL VALENCE (with three levels; pleasant/unpleasant/neutral). The analysis of the ERP data contained two additional within-subject factors related to cortical regions of ANTERIORITY (with two levels; anterior/posterior) and HEMISPHERE (with two levels; left/right). Analyses were carried out separately on each of the four temporal windows and additional *post hoc* tests were computed for multiple comparisons. There were insufficient trials to average EEG signals related to errors (misses and FAs). In all analyses the significance level was set at 5% (two-tailed) with Greenhouse-Geisser corrections for degrees of freedom where necessary.

## 3. RESULTS

### 3.1. Behavioral Data

#### 3.1.1. Valence and Arousal Ratings

An ANOVA on the valence ratings showed significant differences between the three categories (F(2,38) = 310.42; p < 0.001). Multiple comparisons (Bonferroni) *post hoc* tests showed that the mean valence rating was significantly different between neutral and unpleasant (mean difference = 2.39; p < 0.001), between neutral and pleasant (mean difference = 2.14; p < 0.001) and between pleasant and unpleasant (mean difference = 4.45; p < 0.001). Thus, our participants’ valence rating scores were consistent with the IAPS’s standard scores where differences between IAPS and our results were 0.05 for the pleasant, 0.02 for the neutral and 0.33 for the unpleasant (see Table 1).

An ANOVA on the arousal ratings also showed significant differences between the three categories (F(2,38) = 177.30; p < 0.001). Multiple comparisons (Bonferroni) *post hoc* tests showed that the mean arousal rating was significantly different between neutral and unpleasant (mean difference = 3.09; p < 0.001), between neutral and pleasant (mean difference = 1.17; p < 0.001) and between pleasant and unpleasant (mean difference = 1.92; p < 0.001). Our participants’ arousal ratings were consistent with the IAPS’s standard scores for the pleasant (difference = 0.2) and neutral (difference = 0.2), but not for the unpleasant where they gave higher arousal values than the IAPS (difference = 1.2) (see Table 1).

#### 3.1.2. Discrimination Accuracy, Response Bias and Reaction Times

Hits and correct rejection rates for the unpleasant, pleasant and neutral items along with discrimination and response bias indices are shown in Table 2. The Old/new discrimination accuracy (Pr) was affected by emotional valence (F(2,38) = 6.64, p < 0.01). Multiple comparisons (BONFERRONI) revealed that the Pr was smaller to pleasant than neutral (p < 0.05) conditions and also when contrasting pleasant with unpleasant (p < 0.05), but no significant difference emerged between unpleasant and neutral images. The response bias (Br) consistently showed a significant emotional valence effect (F(2,38) = 10.49, p < 0.001). Multiple comparisons (BONFERRONI) revealed that the Br was higher for pleasant than for neutral (p < 0.001) images and for unpleasant than for neutral (p < 0.005) images, but no significant difference emerged between unpleasant and pleasant images.

Reaction Times (RTs) data are shown in Table 2. An ANOVA applied to median RTs revealed a main effect of response type (F(1,19) = 10.30, p < 0.01), emotional valence (F(1,19) = 10.46, p < 0.001) and an interaction between these factors (F(2,38) = 5.99, p < 0.01). Multiple comparisons (BONFERRONI) revealed that hits (old) were identified significantly faster (955 ms) than correctly rejected (1018 ms) images. RTs to unpleasant images were delayed (1007 ms) in comparison with neutral (985 ms) and pleasant (968 ms) images. Further paired comparisons revealed that the response type effect was significantly larger in both unpleasant (t(19) = 4.48, p < 0.001) and pleasant (t(19) = 2.24, p < 0.05) in comparison with the neutral (t(19) = 1.88, p = 0.07).

### 3.2. Electrophysiological Data

**Figure 1:**
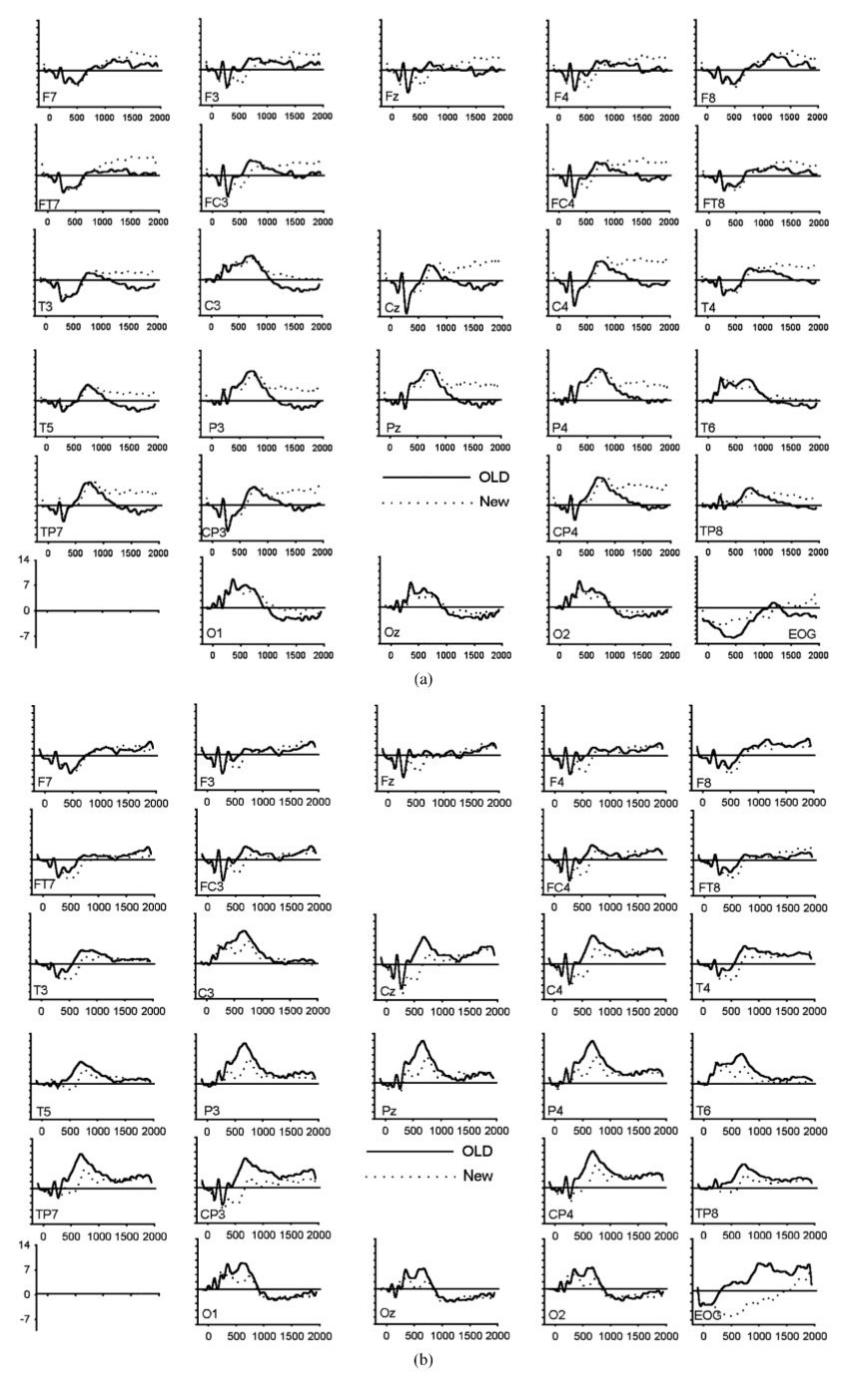

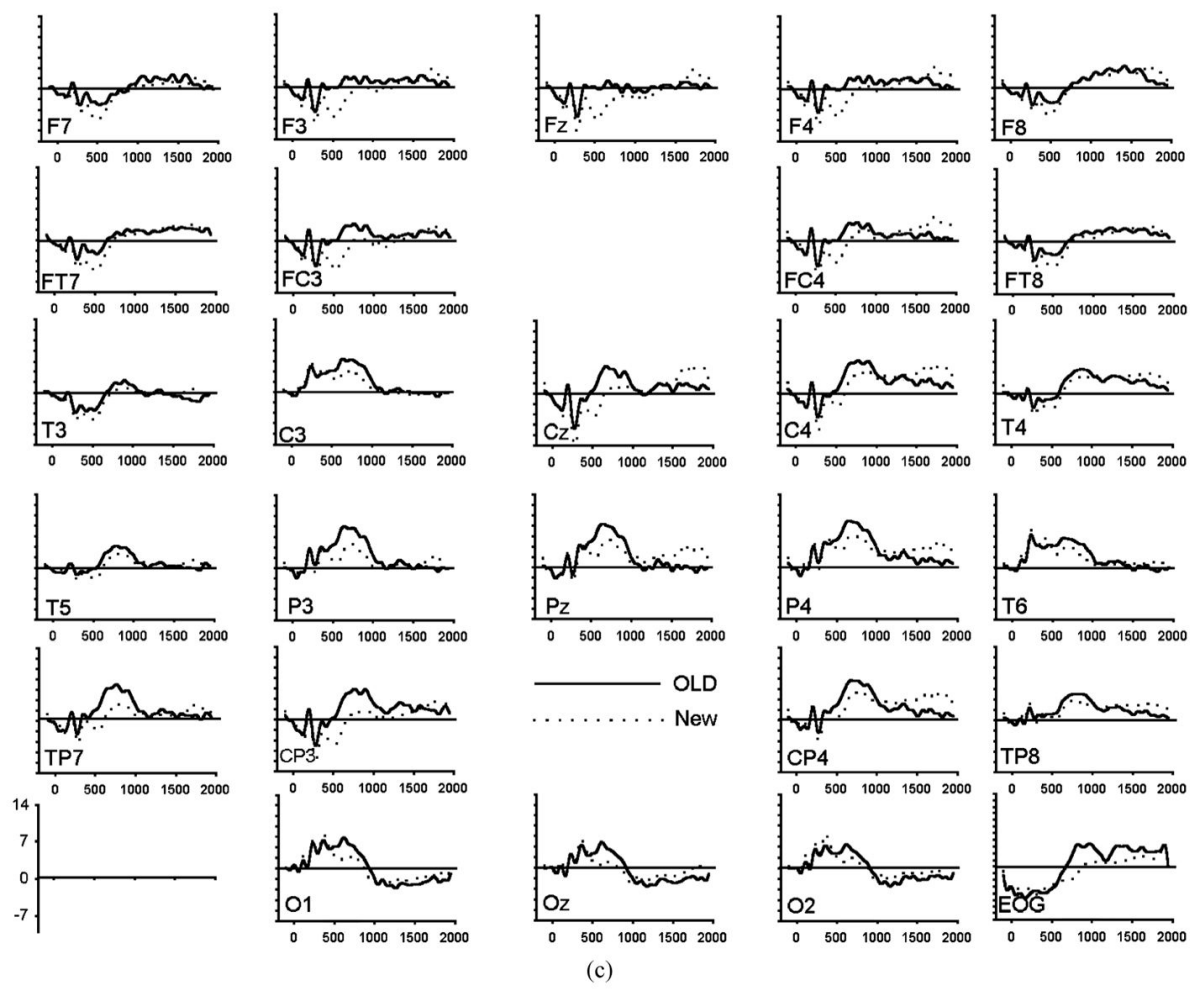
(a) Grand average of ERP waveforms elicited by correctly classified old (bold) and new (dotted) unpleasant images; (b) Grand average of ERP waveforms elicited by correctly classified old (bold) and new (dotted) pleasant images; (c) Grand average of ERP waveforms elicited by correctly classified old (bold) and new (dotted) neutral images.

To assess amplitude differences between conditions, ANOVAs were carried out for each latency window separately. Figure 1 shows the grand average of the ERPs (n = 20) for correctly recognized old and new images for all electrode locations and conditions. Table 3 presents all significant interactions for the four ERP intervals during the test.

#### 3.2.1. Early Old/New Effect (300 – 500 ms)

**Figure 2:**
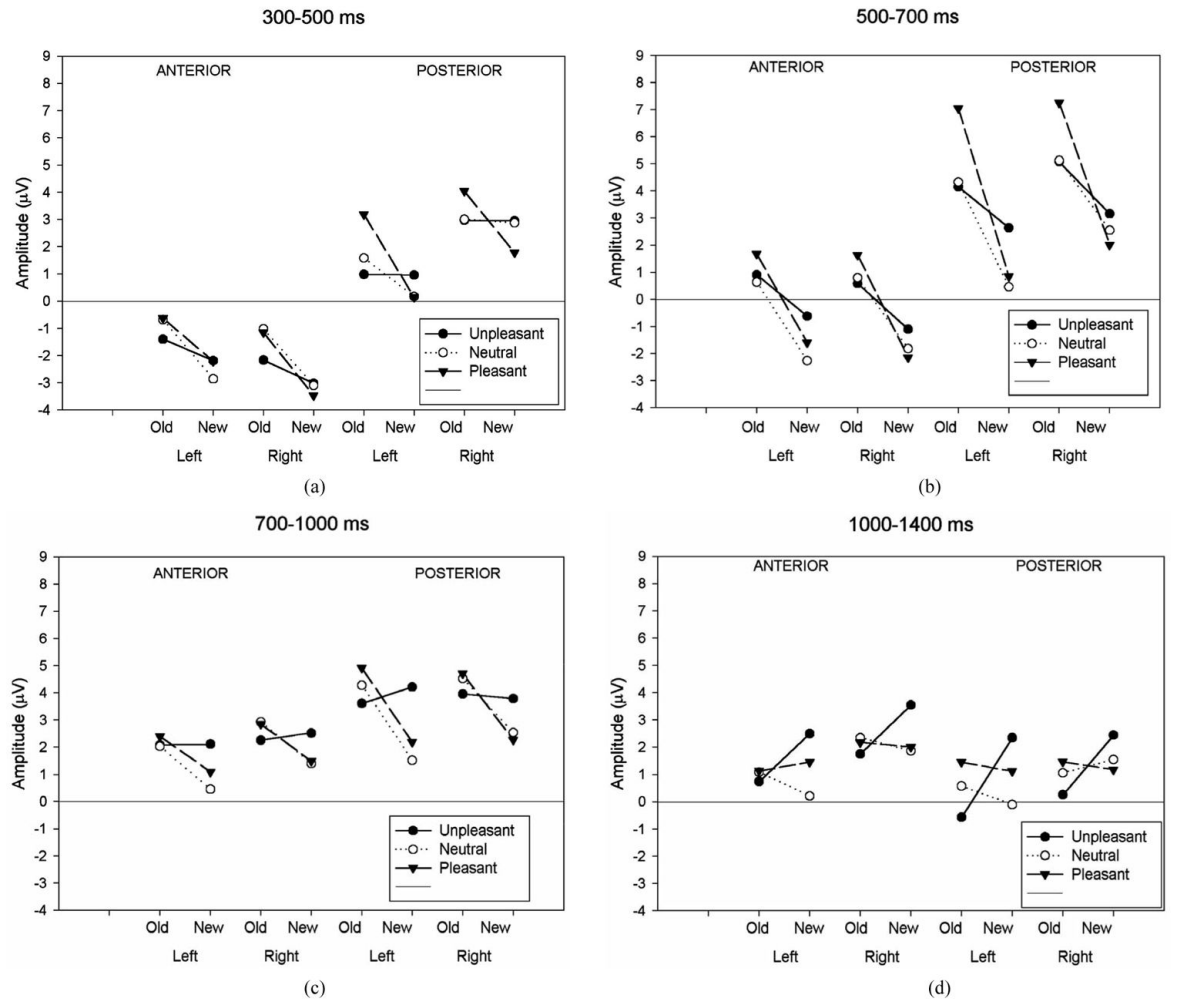
(a) Mean ERP amplitude measured in the four time-windows as a function of anteriority (anterior-posterior), hemisphere (left-right) and response type (old-new) conditions. Old-new effects for neutral images are compared to old-new effects for unpleasant and pleasant images. Illustrations show these comparisons for the 300 – 500 ms; (b) Mean ERP amplitude measured in the four time-windows as a function of anteriority (anterior-posterior), hemisphere (left-right) and response type (old-new) conditions. Old-new effects for neutral images are compared to old-new effects for unpleasant and pleasant images. Illustrations show these comparisons for the 500 – 700 ms; (c) Mean ERP amplitude measured in the four time-windows as a function of anteriority (anterior-posterior), hemisphere (left-right) and response type (old-new) conditions. Old-new effects for neutral images are compared to old-new effects for unpleasant and pleasant images. Illustrations show these comparisons for the 700 – 1000 ms; (d) Mean ERP amplitude measured in the four time-windows as a function of anteriority (anterior-posterior), hemisphere (left-right) and response type (old-new) conditions. Old-new effects for neutral images are compared to old-new effects for unpleasant and pleasant images. Illustrations show these comparisons for the 1000 – 1400 ms.

As can be seen in Table 3 and Figure 2(a), analysis of the early latency period (300 – 500 ms) revealed a significant main effect of response category and anteriority. A significant response type by anteriority by valence interaction was also found. In order to further examine the nature of these interactions, two subsidiary ANOVA was carried out separately on anterior and posterior regions. The first analysis, applied to the anterior region, revealed a response type main effect (F(1,19) = 25.18, p < 0.001) expressed as a more negative amplitude to new ( 2.80 uV) than old ( 1.16 uV) images. The emotional valence factor failed to reach significance at the anterior region. The second analysis, applied to the posterior region, revealed a significant main effect of response type (F(1,19) = 9.55, p < 0.01), hemisphere (F(1,19) = 7.45, p < 0.05) and an interaction between these factors (F(1,19) = 8.61, p < 0.01). This interaction was expressed by a larger response type effect over the left (t(19) = 3.53, p < 0.005) than over the right (t(19) = 2.27, p < 0.05) hemisphere. An interaction between response types and emotional valence was also present (F(2,38) = 3.96, p < 0.05). Further ANOVAs, computed separately for each valence class, showed that the effect of response type was significant only for the pleasant category (pleasant: (F(1,19) = 16.09, p < 0.001; unpleasant: (F(1,19) = 0.001, p = 0.97; neutral: (F(1,19) = 1.19, p = 0.29).

#### 3.2.2. Left Parietal Old/New Effect (500 – 700 ms)

This latency window (500 and 700 ms) is depicted in Figure 2(b) for all conditions. A significant main effect of response type and anteriority along with several interactions involving the hemisphere and the valence factors was shown (Table 3). In order to assess the reliability of the old-new response type effect, elicited by each class of emotional valence, separate ANOVAs were applied to emotional categories separately. The analysis revealed a main effect of response type in each emotional condition (unpleasant: F(1,19) = 5.19, p < 0.05; pleasant: F(1,19) = 33.08, p < 0.001; neutral: F(1,19) = 18.32, p < 0.001). An interaction between response types and anteriority was also present for the pleasant (F(1,19) = 33.08, p < 0.001) and neutral category (F(1,19) = 4.39, p < 0.05), indicating that the old-new effect in the pleasant and neutral category was maximal at posterior regions. A separate ANOVA focusing on the posterior region revealed a significant interaction between response type, valence and hemisphere (F(1.98,37.71) = 8.06, p < 0.001). Separate ANOVAs, calculated for left and right posterior regions, revealed a main effect of response type in both hemispheres (left: F(1,19) = 35.53, p < 0.01; right: F(1,19) = 30.29, p < 0.01) while the emotional valence main effect was only significant in the left hemisphere (left: F(2,18) = 11.03, p < 0.05; right: F(2,18) = 1.22, p = 0.32). The multiple comparison test (BONFERRONI) for the left hemisphere showed a larger positive amplitude in unpleasant compared to neutral (p < 0.05) images and also a larger amplitude between pleasant and neutral (p < 0.05) images, but no differences in amplitude between unpleasant and pleasant (p = 0.32). The interaction between response type and emotional valence was also more pronounced in the left than in the right hemisphere (left: F(2,18) = 11.03, p < 0.001; right: F(2,18) = 5.71, p < 0.05). Figure 2(b) clearly shows the left posterior location of the old-new effect for both pleasant and neutral images and a reduced old-new effect for the unpleasant. Two further ANOVAs administered to the posterior region showed a significant valence effect separately for the new (F(2,18) = 3.73, p < 0.05) and old (F(2,18) = 8.89, p < 0.001) images. Post hoc tests carried out to the new images revealed that the waveforms elicited by unpleasant images were significantly more positive (2.91 uV) compared to the new neutral (1.51 uV) and new pleasant (1.44 uV) images. The inverse relationship was found with the old images. The waveforms elicited by old unpleasant images were significantly less positive (4.62 uV) compared to old neutral (4.73 uV) and old pleasant (7.15 uV) images.

#### 3.2.3. Late Parietal Old/New Effect (700 – 1000 ms)

For the next period (700 – 1000 ms), a significant main effect of response type and an interaction between response type and valence remained significant (Figure 2(c)). As for the previous 500 – 700 ms latency window, the reliability of the old-new response type effect was assessed in each class of emotional valence and separate ANOVAs were computed for the three emotional conditions separately. The ANOVA for the neutral and pleasant revealed a main effect of response type (neutral: F(1,19) = 7.21, p < 0.05; pleasant F(1,19) = 8.85, p < 0.01) while the unpleasant showed no effect of response type (F(1,19) = 0.09, p = 0.76). A further ANOVA carried out to the posterior region showed that the waveforms elicited by new unpleasant images were significantly more positive than those elicited by the new neutral and new pleasant images (F(2,18) = 11.33, p < 0.001) whereas no such effect was present for the ERPs elicited by the three classes of old images.

#### 3.2.4. Late Old/New Effect (1000 – 1400 ms)

For the last time window (1000 – 1400 ms), a main effect of response type and an interaction between response type and emotional valence and between hemisphere and emotional valence was significant (Figure 2(d)). The separate ANOVAs to the neutral and pleasant showed no response type effect (neutral: (F(1,19) = 0.57, p = 0.46; pleasant (F(1,19) = 0.03, p = 0.88), whereas the analysis applied to the unpleasant revealed a significant response type effect (F(1,19) = 7.98, p < 0.05). Further ANOVAs investigating the hemisphere effect in each valence condition showed no hemisphere effects in both emotional categories (unpleasant: (F(1,19) = 1.87, p = 0.19; pleasant (F(1,19) = 0.66, p = 0.43), whereas the analysis applied to the neutral images revealed a significant hemisphere effect with a larger amplitude to the right hemisphere (F(1,19) = 6.44, p < 0.05).

## 4. DISCUSSION

### 4.1. Effect of Emotional Valence on Recognition Memory Performance

We hypothesized that both emotional valence and arousal would have an impact on recognition performances. Our results consistently showed that high arousal emotional images (pleasant and unpleasant) affect recognition performances more than neutral pictures. For instance, the FA rates were significantly greater for both emotionally unpleasant and pleasant images than for neutral images. In other words, while emotional images were correctly identified as old, more frequently than neutral images, about the same proportion of new emotional, relative to neutral images were erroneously identified as old. Likewise, the response bias (Br) was also higher to both unpleasant and pleasant compared to neutral images, meaning that emotional images were classified as old more often than neutral images, whether or not they were old. Consistently, RTs were also affected by the old-new status and followed the same pattern as the FA rates and the Br. As expected, the newly presented images delayed the RTs compared to old images. These RT old-new differences were larger as well, for both emotional categories than for the neutral one.

We can tentatively propose a pragmatic explanation whereby a FA is generated for an uncertain state, reflecting a direct estimate of the probability of a ‘yes’ response under uncertainty [33]. This result then indicates that participants were more likely to say ‘yes’ to emotional than to neutral images when they were not sure about their responses and so the level of FA was greater. Consistent with this hypothesis, our results also showed that emotional pictures required more processing time to be accurately discriminated than neutral pictures. Indeed, the old-new effect was 60% longer in response to unpleasant than to neutral images. This is consistent with previous studies, which found an increase of the old-new effect by 46% [22] and 32% [23] for correctly classified aversive words compared to neutral ones. This was also the case in a similar previous study using emotional pictures recognition, with 36% larger old-new effect in response to to unpleasant than to pleasant [24] Consistent with the FA rates, longer old-new RT differences could reflect this response bias to emotional material if high arousal images (pleasant and unpleasant) involve affect that is more salient and motivating. This could result in a higher response bias toward emotional images due to their higher saliency. McNeely, Dywan, & Segalowitz [34] supported the view that the salience of emotional words can be falsely attributed to familiarity in the context of episodic retrieval. Consistent with these results, our behavioral data suggests that the emotion-induced response bias was mainly affected by the arousal value of a stimulus rather than by its degree of pleasantness (pleasant or unpleasant), favoring the emotional saliency approach. In sum, our results imply that both unpleasant and pleasant emotional images show higher propensity to elicit a response bias than the neutral images, probably because they have more salient attributes.

### 4.2. Effects of Emotional Valence on ERP Manifestation of Old/New Effect

As expected, our results showed three topographically distinct components expressed by 1) an early frontal old new effect arising between 300 and 500 ms, 2) an overlapping late old-new effect maximum over left posterior regions occurring between 300 and 1000 ms and finally, 3) a late component arising between 1000 – 1400 ms. The second aim of the current investigation was to consider whether these three recognition memory stages, as indexed by ERPs, were affected in a similar manner by emotional valence and arousal. The electrophysiological results showed that emotional valence had a clear impact on later parietal old-new effects while leaving intact the early frontal negativity.

#### 4.2.1. Effect of Emotional Valence on the Early Old/New Effect

In the first temporal window (300 and 500 ms post-stimulus), right-frontal amplitude was mediated by the response type (old vs new) but not by the emotional valence of images. This frontal negativity was previously identified, in an old/new paradigm, as a FN400 [35]. According to several investigations, the FN400 old/new effect appears to reflect familiarity recognition rather than conscious recollection (Curran, 1999; Friedman *et al*., 2000; Rugg *et al*., 1998b). Familiarity is defined as the capacity to assess the conceptual similarity between a test item and all study-list information in memory [36]. If we confine our interpretation to the FN400 *per se*, our results imply that emotional valence has no significant effect on the assessment of familiarity. This is consistent with an earlier study which found that this frontal old/new effect elicited by negative words was indistinguishable from those elicited by neutral items [22]. Our results also showed that the insertion of pleasant images, among unpleasant and neutral images, elicited a comparable impact on the assessment of familiarity.

#### 4.2.2. Effect of Emotional Valence on the Left Parietal ERP Old/New Effect

Emotional valence had a marked effect on the left posterior deflection, where the old-new effect is usually largest. The interaction of emotional valence with response type, partially overlapping with the earlier frontal negativity, was apparent by 300 ms post-stimulus and extended until 1000 ms. This large positive deflection showed a robust left parietal old/new effect, and was prominent in both pleasant and neutral condition, but almost absent in the unpleasant condition. According to the expected scalp distribution and latency timing, this positive complex is analogous to the classical Late Positive Component (LPC) observed during old/new memory experiments with non-emotional words (Curran, 1999) and pictures (Curran *et al*., 2003) often thought to reflect conscious recollection. This idea is also consistent with previous ERP studies of recognition memory, which reported a reduced parietal effect, in the same latency range, elicited for negative words in comparison with that elicited in response to neutral words (Maratos *et al*., 2000; Maratos, Dolan, Morris, Henson, & Rugg, 2001). With emotional images, Gasbarri *et al*. [37] and Glaser *et al*. [24] reported reduced left lateralized activity over the parietal area in women, in response to unpleasant pictures.

As with our results, these differences between neutral and negative material in the magnitude of the left parietal effect were attributable to the increased amplitude elicited by new unpleasant items relative to old ones. The amplitude of the LPC, within the context of the old/new paradigm, has often been reported to be sensitive to the recollection of specific details and/or depth of processing [11,38]. Furthermore, earlier investigations showed that unpleasant pictures elicit more marked physiological responses than neutral or pleasant pictures during the recognition of emotional images [39–41]. For instance, an ERP study of the subsequent memory effect also recorded a LPC, between 400 and 600 ms, sensitive to both arousal and valence over central electrodes [29]. These results suggest that unpleasant emotional information has privileged access to processing resources through its activation of cerebral structures, responsible for improved memory formation. Indeed, the left parietal old/new effect is considered to reflect episodic memory retrieval associated with the medial temporal lobe system including its projection into prefrontal and parietal areas [11, 42–45]. In the context of the current study, similar neural pathways are probably active during conscious recollection in all types of emotions but are selectively more activated especially in an unpleasant situation. Thus, emotional tasks with explicit cognitive demand share some of those networks and enhance the processing of threatening information [46].

#### 4.2.3. Effect of Emotional Valence on the Late Old/New Effect

Finally, the analysis of the later component (1000 – 1400 ms) revealed an interaction between old-new response type and emotional valence. However, the nature of the interaction was expressed differently from that observed with earlier components. We found that unpleasant images elicited an inverted old/new effect over posterior areas, whereas new images elicited larger amplitude than old ones. A component within this latency range is thought to reflect a process of post-retrieval monitoring appearing after response completion [32]. Logically, new items had not been seen during the study episode and no episodic mnemonic information could have been retrieved. So, this old/new effect could not reflect retrieval success, suggesting instead that it reflects processes that monitor the retrieval of information, instead of itself constituting a manifestation of successful retrieval [19, 47]. After conscious recollection, a post retrieval monitoring system could re-evaluate the content of the correctly retrieved information in order to establish if it represents an accurate episode from the study phase [48]. It was recently argued that this posterior component could be related to a retrieval mechanism that reconstructs the prior episode when task-relevant features such as color and contexts in which it was studied require sustained monitoring [49]. In the context of our study, we obtained a very low level of FA rate for all categories, so we can’t confidently relate our results to this hypothesis. But despite the lack of evidence that this late component varies according to response type in pleasant and neutral conditions, we can tentatively propose that larger sustained amplitudes to new unpleasant images are a reflection of additional monitoring resources, which reevaluate aversive pictures not yet encoded in memory. According to past studies in memory of emotional images, the valence activates selective attention, whereas arousal is elicited by stimulus motivational qualities engaging attentional resources that contribute to memory encoding [29].

Based on the fact that the emotional arousal is similar across pleasant and unpleasant categories, we can further propose that the valence dimensions remain particularly important after response completion. At that point in time, the valence is monitored as an important attribute contained in the image. This monitoring must continue after the response completion in order to establish that the unpleasant images are properly encoded. This type of double-check would be, thus, less essential in an emotionally pleasant or neutral context.

## 5. CONCLUDING COMMENTS

The first aim of the current study was to investigate whether recognition performance was affected by the presentation of emotional images controlled for valence and arousal ratings. Our behavioral performance results were comparable to earlier reports of recognition memory effects obtained with emotional words and pictures. Higher rates of FA along with a greater response bias and larger old-new RT differences were found for both unpleasant and pleasant images as compared with the neutral images. Thus, the recognition bias was mainly affected by the arousal of a stimulus rather than by the emotional valence (pleasant or unpleasant). The second goal was to consider whether recognition memory stages were affected by emotional arousal or emotional valence. Our results showed that the electro-cortical activity was modulated by emotional valence at different points in the processing stream. For instance, findings for the early frontal negativity showed that similar familiarity-based processes were present in all emotional conditions, while later processing closer to the response times and involving conscious recollection and post-retrieval monitoring seems particularly affected by the presentation of unpleasant images.

In summary, the current findings further support the view that arousal level cannot entirely account for the impact of emotions on stages of recognition memory and that emotional valence also plays a role in the recognition bias. So, it may be that a common underlying component in the neural network mediates both emotional dimensions and affects at least two memory stages along the processing stream, that is to say, conscious recollection and post-retrieval monitoring.

## Figures and Tables

**Table 1:** Comparison of the mean “valence” and “activation” between IAPS female norms (Center for the study of emotion and attention [CSEA-NIMH], 1998) and our participants evaluations.

		IAPS female normative data		*Current study* (*n* = 20)	

Valence	Response Types	Valence	Arousal	Valence	Arousal
Pleasant	OLD	7.9	5.2	7.5	6.6
	NEW	7.6	5.6	7.3	6.7
Unpleasant	OLD	2.9	4.3	2.9	4.9
	NEW	2.8	4.8	2.8	4.5
Neutral	OLD	4.9	3.7	4.9	3.4
	NEW	5.6	3.7	5.6	3.7

Note: Valence rating range are from 1 = very unpleasant, 5 = neutral, 9 = very pleasant.

**Table 2:** Probabilities of correct responses to old (hits) and new (correct rejection) items, Reaction Times, and measures of old and new discrimination, false alarms and response bias.

	Hits		Correct rejection		Performance measures		

Image types	*p* (*old*)	*RT* (*SD*)	*p new*	( *RT SD*)	Pr	FA	Br
UNP	0.87 (0.12)	978 (161)	0.91 (0.08)	1035 (200)	0.78	0.09	0.41
PL	0.80 (0.14)	920 (163)	0.89 (0.05)	1016 (200)	0.70	0.11	0.37
Neu	0.79 (0.13)	967 (160)	0.96 (0.07)	1002 (191)	0.75	0.04	0.16

Note. Standard deviations are given in parentheses. Reaction Times (RT) are displayed in milliseconds. UNP: Unpleasant; PL: Pleasant; Neu: Neutral; Pr: discrimination accuracy; FA: False alarms; Br: response bias.

**Table 3:** Summary of ANOVAs results during the four latency windows. Significant interaction effects are reported^a^.

	300 – 500 ms	500 – 700 ms	700 – 1000 ms	1000 – 1400 ms
Old/New (ON)	F = 17.99	F = 34.74	F = 6.31	
Valence (V)				
Anteriority (A)	F = 15.41	F = 15.30		
Hemisphere (H)				
ON × V		**F = 5.22**	**F = 5.24**	**F = 4.84**
ON × A		**F = 9.28**		
ON × H				
V × A				
V × H		**F = 4.8**	**F = 4.81**	**F = 5.65**
ON × A × H	F = 20.06	F = 7.9		
ON × A × V	**F = 5.31**	**F = 5.56**		
ON × H × V		**F = 3.52**		
ON × H × A × V		**F = 4.59**	**F = 4.90**	**F = 3.57**

a Significant F values of interactions involving the factors of valence and response category are shown in **bold**.
